# Transcriptomic Analysis to Understand the Nitrogen Stress Response Mechanism in BNI-Enabled Wheat

**DOI:** 10.3390/ijms26104610

**Published:** 2025-05-12

**Authors:** Chandra Nath Mishra, Sushma Kumari Pawar, Swati Sharma, Arun Thakur, Sabhyata Sabhyata, Shubham Mishra, Satish Kumar, Om Prakash Gupta, Arun Kumar Joshi, Ratan Tiwari

**Affiliations:** 1 ICAR-Indian Institute of Wheat and Barley Research, Karnal 132001, India; sushpawar15.phd@gmail.com (S.K.P.); swatisharma194.ss@gmail.com (S.S.); thakur898arun@gmail.com (A.T.); sabhyatageer27@gmail.com (S.S.); anupam.mishra909@gmail.com (S.M.); satish.kumar8@icar.gov.in (S.K.); op.gupta@icar.gov.in (O.P.G.); ratan.tiwari@icar.gov.in (R.T.); 2 International Maize and Wheat Improvement Center (CIMMYT), NASC Complex, DPS Marg, New Delhi 110012, India; a.k.joshi@cgiar.org; 3 Borlaug Institute for South Asia (BISA), NASC Complex, DPS Marg, New Delhi 110012, India

**Keywords:** BNI, nitrification inhibition, transcriptomics, unigene, wheat

## Abstract

A comparative transcriptomic analysis was conducted for the nitrogen-efficient (BNI-Munal) and derivative parent Munal wheat genotypes to unravel the gene expression patterns across four nitrogen levels (0%, 50%, 75%, and 100%). Analyzing the genes of BNI-enabled wheat helps us understand how they are expressed differently, which heavily influences BNI activity. Grain yield and 1000-grain weight were higher in BNI Munal than in Munal. All the other traits were similar in performance. Varying nitrogen dosages led to significant differences in gene expression patterns between the two genotypes. Genes related to binding and catalytic activity were prevalent among molecular functions, while genes corresponding to cellular anatomical entities dominated the cellular component category. Differential expression was observed in 371 genes at 0%N, 261 genes at 50%N, 303 genes at 75%N, and 736 genes at 100%N. Five unigenes (three upregulated and two downregulated) were consistently expressed across all nitrogen levels. Further analysis of upregulated unigenes identified links to the NrpA gene (involved in nitrogen regulation), tetratricopeptide repeat-containing protein (PPR), and cytokinin dehydrogenase 2. Analysis of downregulated genes pointed to associations with the *Triticum aestivum* 3BS-specific BAC library, which encodes the NPF (Nitrate and Peptide Transporter Family) and the TaVRN gene family (closely related to the TaNUE1 gene). The five unigenes and one unigene highlighted in the Kyoto Encyclopedia of Genes and Genomes (KEGG) pathways were validated in Munal and BNI Munal. The results obtained will enhance our understanding about gene expression patterns across different nitrogen levels in BNI wheat and help us breed wheat varieties with the BNI trait for improved NUE.

## 1. Introduction

Wheat stands as a vital staple food globally, meeting the energy and protein needs of densely populated countries and regions such as India, China, and the European Union. In 2023-24, these areas led the world in wheat consumption. In the 2022-23 period, India alone cultivated wheat over an area of approximately 31.9 million hectares (mha) (https://pib.gov.in/PressReleaseIframePage.aspx?PRID=1906880, accessed on 20 January 2025), while globally, wheat accounted for 220 mha of cultivated area (https://www.statista.com/statistics/272536/acreage-of-grain-worldwide-by-type/ accessed on 12 December 2024). This extensive cultivation has led to increased nitrogen application worldwide. The conversion of soil nitrogen into nitrates, followed by their leaching, harms ecosystems and human health [1,2]. Excessive nitrogenous fertilizer use also results in the release of nitrous oxide (N_2_O) gas into the atmosphere via denitrification [3]. Additionally, runoff from fields into lakes and rivers can cause nitrogen accumulation and algal blooms, disrupting aquatic ecosystems [4].

Such issues arise because wheat typically has low nitrogen-use efficiency (NUE), meaning it does not fully utilize the nitrogen applied during cultivation [5]. Wheat cultivation accounts for one-fifth of the world’s industrial nitrogen fertilizer use, with a modest NUE of 33%, unchanged for two decades [6,7]. This is where biological nitrification inhibition (BNI) offers a solution. This natural ability allows plants to release compounds from their roots into the rhizosphere, curbing soil nitrifying bacteria activity. This regulation slows nitrate formation and balances soil nitrogen forms (both NH_4_^+^ and NO_3_^2−^), with ammonium ions assimilation being about 40% less energy intensive than nitrate ion assimilation [8]. Wheat demonstrates more robust growth when using ammonium ions over nitrate ions [9,10]. Therefore, breeding and development of BNI-enabled wheat varieties can reduce fertilizer consumption, reduce nitrogen pollution, and contribute to overall soil health [11].

Researchers worldwide are focused on breeding wheat varieties with enhanced BNI capabilities for enhanced NUE [12]. There have been several successful attempts at transferring the BNI trait from *Leymus racemosus* (short arm of 3B chromosome) into elite wheat cultivars like Chinese Spring, Munal, and Roelfs. Developed at the Japan International Research Centre for Agricultural Sciences (JIRCAS), Tsukuba, Japan, in collaboration with the International Maize and Wheat Improvement Centre (CIMMYT), Mexico, BNI-Munal showed suppression of soil nitrifying microbes, resulting in low rates of nitrogen leaching and nitrous oxide emissions. In independent studies conducted by [12] in Japan, Ref [13] in Mexico, and Ref [14] in India, similar yield potentials were reported in BNI-isogenic lines derived from ROELFS and Munal and the mechanism of establishment of the BNI trait through soil dynamics is well established.

In the present investigation, we explore the transcriptomic aspect of the BNI trait to identify genes that are upregulated and downregulated under varying soil nitrogen levels. The key advantage of transcriptomic studies is the identification of molecular markers and candidate genes involved in BNI and NUE capabilities, leading to wheat varieties that perform better in nutrient-limited environments.

## 2. Results

### 2.1. Performance of the Genotypes Based on Agro-Morphological Traits

An analysis of variance revealed that traits like flag leaf length, 1000-grain weight, and different nitrogen treatments were significant sources of variation (Table 1). For plant height, tillers per plant, and grain yield, treatment under different nitrogen doses proved to be a significant cause of variation among those traits. Mean agro-morphological data recorded during the years 2023 and 2024 in all the nitrogen management levels are given in the Supplementary Materials Table S1.

**Figure 1:**
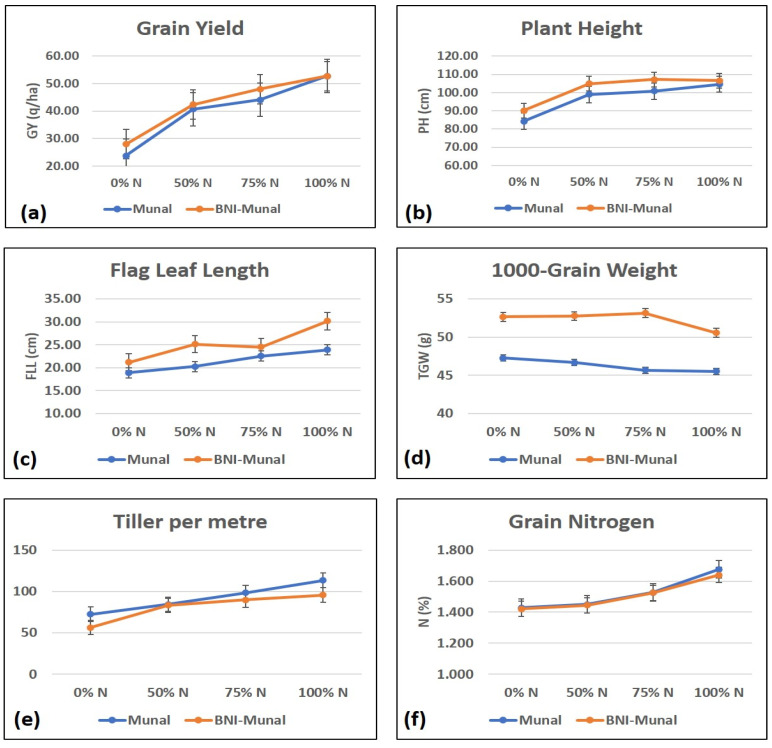
Graphs showing the difference in trait values ((**a**): grain yield; (**b**): plant height, (**c**): flag leaf length; (**d**) 1000 grains weight; (**e**): number of tillers per meter; (**f**): grain nitrogen content) recorded for BNI Munal (orange line) and Munal (blue) line. The dots represent the average value of the trait for both the years, viz., 2023 and 2024.

The graphs (Figure 1) plotted with traits and nitrogen levels revealed that grain yield (q/ha) was higher in the case of BNI-Munal at 0%, 50%, and 75% nitrogen levels but was almost similar at the 100% N-level. The maximum difference in yield was observed at 0% and 75% nitrogen levels. For 1000-grain weight, BNI-Munal exhibited a significantly higher value than the Munal counterpart at all the nitrogen levels. However, 1000-grain weight showed a slight dip from 75%N to 100%N. This can be attributed to the fact that an increase in wheat yield can be up to a certain extent of nitrogen application because N is an essential constituent of chlorophyll and dry matter accumulation. The effectiveness of nitrogen fertilizer application diminishes after a specific dose and can have the opposite effect on the grain yield and its related traits [15]. For tillers per meter, maximum variation was observed at 0% and 100% nitrogen levels and minimum at 50%. Differences among flag leaf lengths were observed to be maximum at 50% and 100% nitrogen levels and minimum at 0% and 75%. The nitrogen levels in grain were very similar at all the nitrogen levels, exhibiting no significant difference between the genotypes.

### 2.2. RNA Extraction and Quantification

Following the quality control pass report, all eight wheat samples, each exposed to different nitrogen dosages, were included in the study. The sample yields varied between 12 μg and 41.5 μg, which were used for downstream RNA-seq analysis. RNA quantification was performed using the Qubit 4.0 Fluorometer, and the results of the gel image (Supplementary Materials Figure S1) and quality check (QC) report are presented in Supplementary Materials Table S2.

### 2.3. Library Construction

Utilizing the Illumina NovaSeq 6000 platform, we generated 95.54 Gb of 2 × 150 bp paired-end (PE) reads from eight samples. These samples were derived from two genotypes across four treatments: N0, N50, N75, and N100. The average library sizes for samples 1 through 8 were 344 bp, 356 bp, 441 bp, 445 bp, 449 bp, 492 bp, 433 bp, and 501 bp, respectively. The sequencing process on the NovaSeq 6000 produced approximately 10–12 GB of data per sample, as detailed in Supplementary Materials Table S3.

A total of 55,668 differentially expressed genes (DEGs) were observed at the 0% nitrogen level. Among these, 371 had significantly differential expression (298 upregulated and 73 downregulated). In comparison, under 100% nitrogen conditions, 52,732 genes were differentially expressed, with 736 being significant DEGs (446 upregulated and 290 downregulated). At 50% nitrogen level, 36,605 DEGs were expressed, with 261 being significant (230 upregulated and 31 downregulated). At 75% nitrogen levels, there were 35,517 DEGs, of which 303 were significant (277 upregulated and 266 downregulated). The transcriptome analysis revealed a substantial difference in adaptive response between N-efficient (BNI-Munal) and N-inefficient (Munal) genotypes, evidenced by their distinct gene expression patterns as the nitrogen dosages varied (Table 2).

### 2.4. Transcript Profile of the RNA-Seq Data

**Figure 2:**
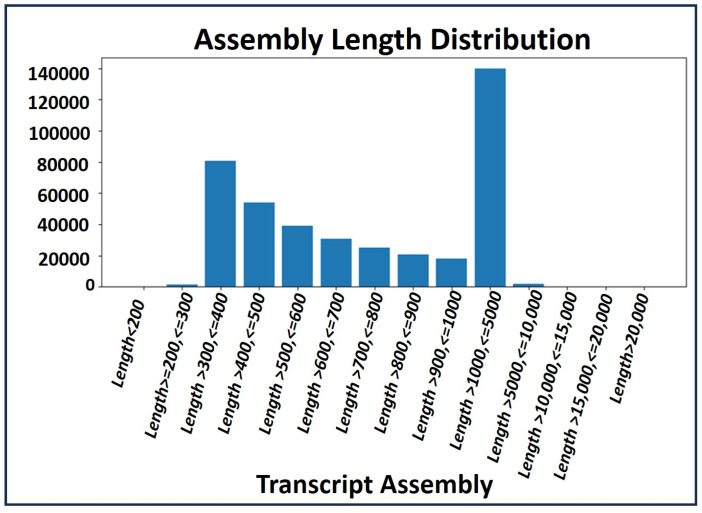
Distribution of transcript assembley length.

A de novo assembly [16] was conducted using high quality (HQ) clean reads to create a comprehensive common assembly for annotation and sample differential expression analysis. Transcript profiles of the RNA-seq data were analyzed by calculating the read fragments per kilobase per million mapped reads (FPKM). The total transcript length generated for gene annotation and differential expression analysis was 413,972,009. The distribution of transcript assembly lengths is illustrated in Figure 2.

### 2.5. Gene Prediction and Differential Gene Expression Study

**Figure 3:**
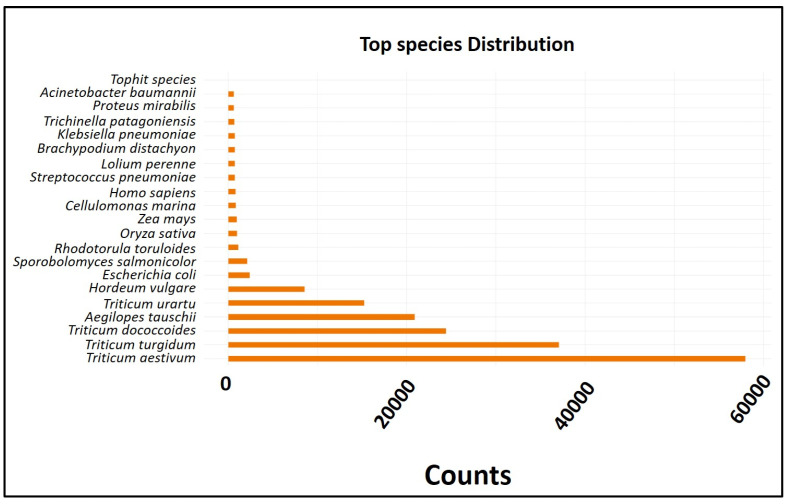
The top-hit species distribution revealed that the majority of the hits were against *Triticum aestivum* and *Triticum turgidum*.

The analysis results are provided together with the normalized counts per million mapped reads (CPM) for each participating sample (Figure 3). According to the top-hit species distribution, *Triticum aestivum* and *Triticum turgidum* were the primary targets, with 300,514 unigenes receiving hits. In total, 300,514 unigenes were identified using the BLAST v. 2.3.13+ database. Each unigene sequence was simultaneously analyzed for similarity against the Uniprot (109,229 hits), KOG (58,795 hits), and Pfam (56,033 hits) databases using BLASTP with an e-value cutoff of 10^−5^.

**Figure 4:**
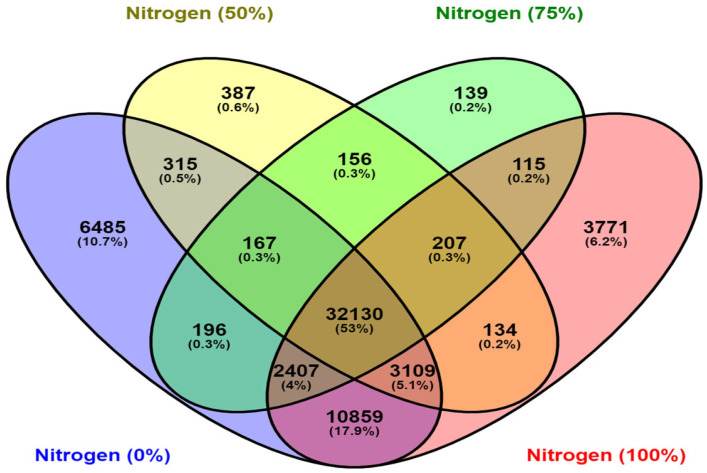
Venn diagram for annotated unigenes in all four DGE comparisons.

Comparative analysis of four DGEs revealed common differentially expressed unigenes (32,130) under varying nitrogen dosage levels, as shown in the Venn diagram (Figure 4).

**Figure 5:**
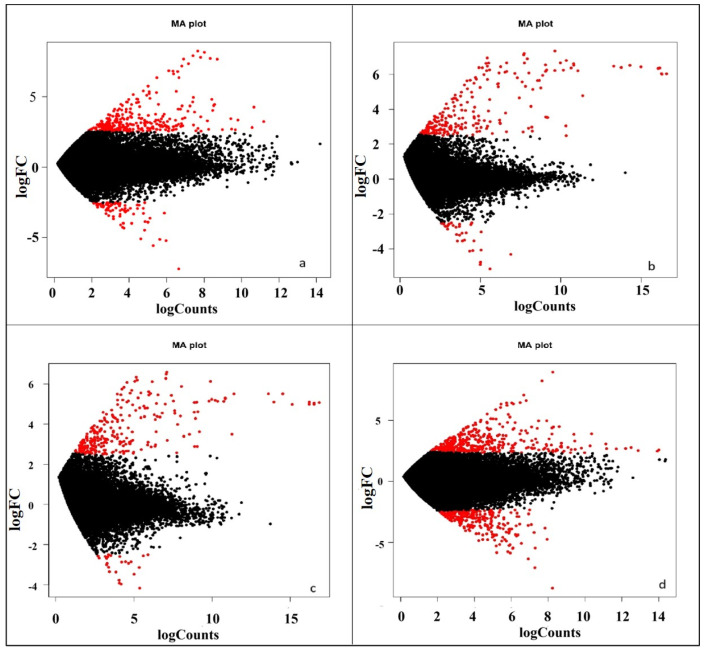
MA-Plot showing comparative differential gene expression under different nitrogen management levels, viz., (**a**) 0% N, (**b**) 50% N, (**c**) 75% N, and (**d**) 100% N. The M-value (y-axis) represents the log2 fold change in expression, and the A-value (x-axis) represents the average log2 expression. Significantly differentially expressed genes (FDR < 0.05) are shown in red while black dots are non-significant.

By transforming the data into M (log ratio) and A (mean average) scales, the graph (Figure 5) illustrates the variations between measurements taken from two samples under different nitrogen levels. This is an effective tool for visualizing the relationship between gene expression levels and the contrasting conditions for differential gene expression. In the plot, the red dots indicate significant differential expression, while the black dots near the (0,0) mark denote non-significant expression. At 0% nitrogen application, there were significantly more upregulated genes than downregulated ones. Similar expression patterns were observed at 50% and 75% nitrogen levels. However, at 100% nitrogen, the numbers of significantly upregulated and downregulated genes appeared somewhat similar. The plots also demonstrate that at 50% and 75% nitrogen, the expression levels of significantly upregulated and downregulated genes are consistent.

**Figure 6:**
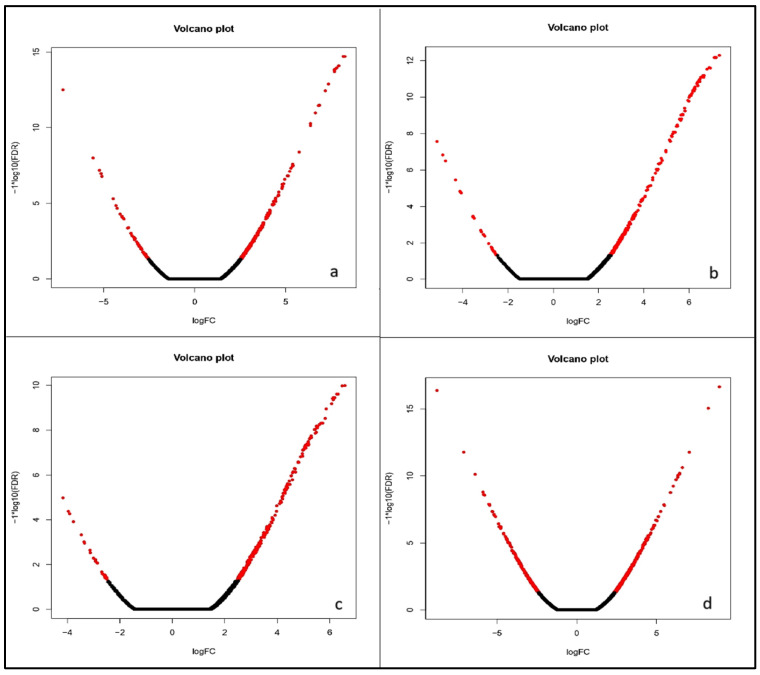
Volcano plot displaying comparative differential gene expression in all four nitrogen ((**a**): 0% nitrogen; (**b**): 50%nitrogen; (**c**): 75% nitrogen; (**d**): 100%nitrogen) management levels. The x-axis represents the log2 fold change and the y-axis represents (−1)^n^log10(FDR) explaining statistical significance and direction of change (n = 1 for downregulated features; n = 0 for upregulated features that yields +log10(FDR) for upregulated and-log10(FDR) for downregulated. Red dots indicate genes with adjusted *p* < 0.05 and absolute log2 fold change >1 while black dots represent non-significant values.

The “volcano plot” maps out expressed genes using biological and statistical significance. Figure 6 represents the volcano plots for all four comparisons. These plots show that at 0%, 50%, and 75% dosage level, there are more significantly upregulated genes than downregulated ones, as indicated by the red dots. However, at 100% dosage level, the number of significantly upregulated and downregulated genes are very similar (Figure 6). Notably, at 50% and 75% nitrogen levels, the highest number of significantly upregulated DEGs were observed with high expression levels.

Across all four nitrogen management levels, five unigenes were consistently identified as significantly expressed, comprising three major upregulated and two significant downregulated unigenes (Table 3). Specifically, Unigenes_153540 and Unigenes_153768 were significantly downregulated, while Unigenes_141059, Unigenes_162379, and Unigenes_222562 were frequently upregulated.

The BLASTP database showed a 100% query cover with the *Triticum aestivum* genome, which encodes the NRT1/PTR FAMILY 2.13-like protein (accession XP_044353808.1) in wheat. The downregulated Unigene_153540 transcript sequence, when searched on the standard nucleotide BLAST with the Nucleotide Collection (nr/nt) database setting, revealed a 100% similarity, 70% query coverage, and an E-value of 1.3 with the low molecular weight (LMW) beta- and gamma-gliadin proteins, which belong to the prolamine super family (Accession no. MG560141.1).

With a 100% query coverage, hit similarity of 97.54%, and an E-value of 1.6 with the *Triticum aestivum* genome (accession id AAW73220.1), the downregulated unigene code for TaVRN-A1 (transcript_153768) is a key regulator of the vernalization response in wheat, affecting flowering time and crop development. Studies have shown a direct association between vernalization and NUE in wheat. The epigenetic regulation and interaction with other vernalization genes ensure optimal flowering time for successful reproduction and high yields.

The upregulated transcript_162379 sequence codes for cytokinin dehydrogenase 2 with 100% query cover, 91.49% similarity, and an E-value of 0 (XP_044355953.1). CKX2 is important in regulating cytokinin levels in wheat, influencing key developmental processes, stress responses, and, potentially, grain yield [17]. Manipulating CKX2 could be valuable for improving wheat productivity and resilience.

*Triticum aestivum* cover for the elevated PPR protein unigene code (XP_044339860.1), transcript_141059, was 100%, with a 99.36% identity and an E-value of 0. At5g57250, also called AtPPR_At5g57250 or PPR protein (AtPPR), is a pentatricopeptide repeat-containing protein that resembles mitochondria and is found in *Arabidopsis thaliana* [18]. PPR proteins are typically involved in regulating RNA processing and stability and are often located in mitochondria and plastids, organelles involved in energy production and photosynthesis.

With a 66% query coverage and 100% similarity and E-value 1.6, the upregulated transcript_222562 is identified as the CRB-INRA-CFD-3358 nodulin-related protein (NrpA) gene, exon 2, and incomplete cds (EF114997.1). The NrpA gene (CRB-INRA-CFD-3358, EF114997.1) in wheat likely encodes a nodulin-related protein that may play a role in nitrogen uptake, stress response, or other metabolic and developmental processes [19]. Further research is needed to precisely determine its function in wheat.

### 2.6. Gene Ontology

**Figure 7:**
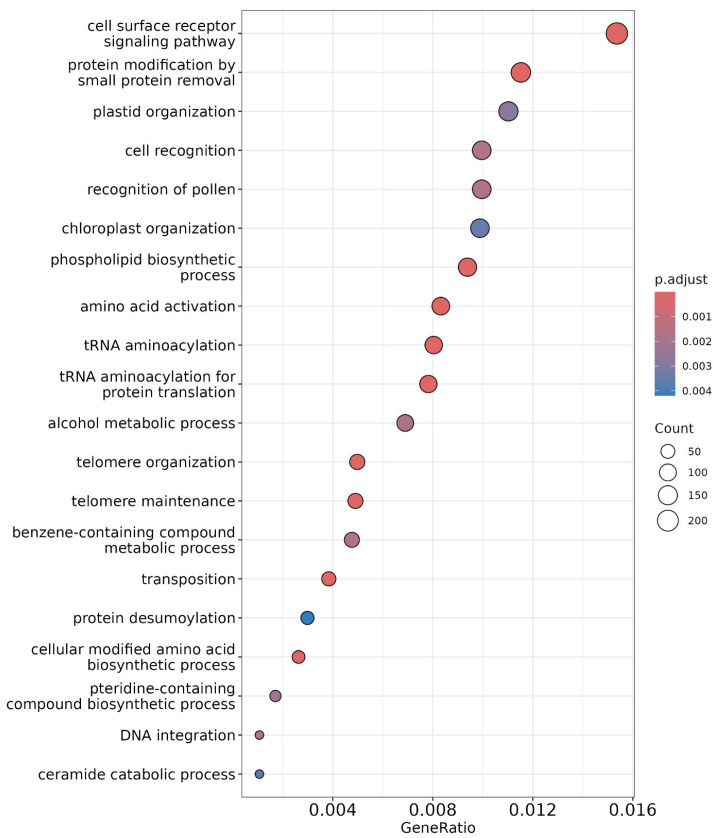
Dot plot representing enrichment analysis of significant GO terms, with the gene ratio on the *x*-axis indicating the proportion of genes associated with each term. The *y*-axis lists the enriched biological processes. Dot size corresponds to the number of genes, while color represents the significance level according to adjusted *p*-values (adj. *p*-value < 0.05).

Gene Ontology (GO) enrichment analysis was performed to identify significantly enriched biological processes using Blast2GO 6.0 software. The results are visualized using a dot plot (Figure 7). Enrichment was determined using Fisher’s exact method and multiple testing using Benjamini Hochberg’s method to reduce the likelihood of false discoveries. The most significantly enriched terms included ‘cell surface receptor signalling pathway’ followed by ‘protein modification by small protein removal’ and ‘plastid organization’ [20]. Cell surface receptors akin to receptor-like kinases and nitrate transporters perceive external nitrogen availability and initiate a signalling cascade for the regulation and uptake of nitrogen in nitrate or ammonium form, and in the case of BNI, the compounds inhibit ammonium-oxidizing bacteria that are exuded from the roots, like methylated flavonoids in wheat [21,22].

### 2.7. KOG Classification and Comparative Gene Annotation

**Figure 8:**
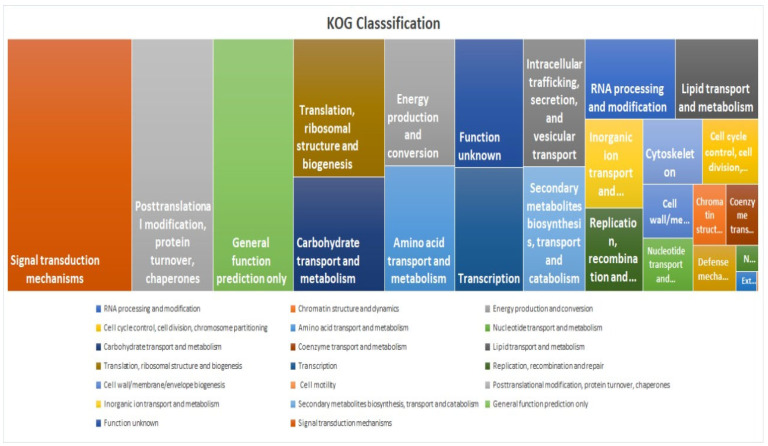
Tree map depicting the KOG classification for unigenes based on their predicted function.

KOG analysis showed that the “Signal transduction mechanisms” category had the highest enrichment, indicating a significant representation of genes involved in cellular communication, environmental sensing, and regulatory pathways (Figure 8). This is followed by “Post-translational modification, protein turnover, and chaperones”, which includes genes essential for protein folding, stability, degradation, and cellular stress responses (Figure 8). The Pfam study identified “Pkinase_Tyr (2982 occurrences)” as the most prevalent domain associated with tyrosine kinase activity, which plays a crucial role in phosphorylation-based signalling pathways. Other abundant domains include “NB-ARC (970 occurrences)”, linked to nucleotide-binding and disease resistance, “zf-RVT (717 occurrences)”, associated with reverse transcriptases, retroviral replication, and mobility of retrotransposons, “p450 (640 occurrences)”, involved in xenobiotic metabolism, playing a role in detoxification and biosynthetic processes, “Pkinase (450 occurrences)”, representing general protein kinases, especially serine/threonine kinases, “RRM_1 (418 occurrences)”, an RNA-recognition motif which plays a role in RNA binding for processing, stability, and translation, “MULE (398 occurrences)”, related to Mutator-like transposable elements contributing to evolution and genetic diversity, “zf-RING_2 (386 occurrences)”, a zinc-finger domain involved in ubiquitination, tagging substrates for proteasomal degradation “PPR_2 (376 occurrences)”, which participated in RNA editing, and “Rx_N (362 occurrences)”, commonly found in plant disease resistance proteins for recognizing pathogen effectors. For comparative unigene annotation, an in-house script was used to create a Venn diagram (Supplementary Materials Figure S2) involving the NR, Uniprot, Pfam, and KOG databases to identify commonly differentially expressed proteins (35551).

**Figure 9:**
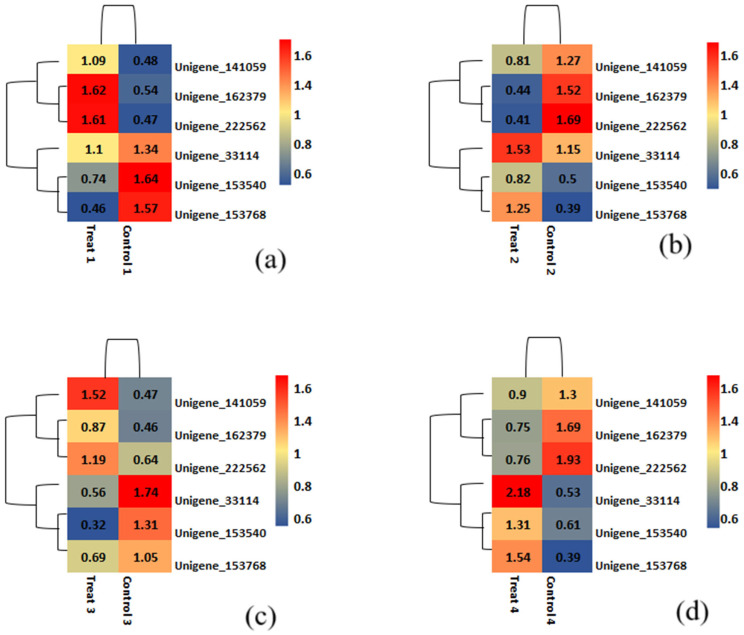
A heatmap representing the most significant differentially expressed genes in both samples was plotted using log10 of the normalized read count values (CPM). The color tone towards red on the heat scale signifies upregulation, while color tone towards blue signifies downregulation, and the intensity of the color represents the magnitude of expression. (**a**) 0% N level; (**b**) 50% N level; (**c**) 75% N level; (**d**) 100% N level.

The heatmap for differentially expressed genes under varying nitrogen dosages is shown in Figure 9, where red indicates expression levels, while blue indicates significantly reduced levels. Six unigenes identified as significantly co-regulated across four comparisons are presented in Figure 9. In the heatmap comparison 1 differentiating Munal (Control) at 0% dosage and BNI Munal (Test) at 0% dosage, Unigene_153768 transcript expression appears to be significantly downregulated. For comparison 2 (at 50% nitrogen dosage), the Unigene_153540 transcript was significantly downregulated. This unigene transcript matched with *protein argonaute 1C-like isoform X1*, but our BLASTN search using the same unigene sequence revealed a relation to the Nitrate/peptide Transporter Family (NRT/PTR1).

Regarding co-upregulated unigenes, transcripts of unigene 162379 and Unigene_222562 showed significantly increased expression levels. The former was a BLASTN hit against a hypothetical protein CFC21_095099, while the latter matched an uncharacterized protein LOC123496932. However, our functional annotation search linked them to *cytokinin dehydrogenase 2* and *nodulin like protein NrpA*, respectively. Both unigenes showed significant co-upregulation in treatment 4 (at 100% nitrogen dosage). Although all mentioned unigenes showed significant differential expression in all four nitrogen regimes, they were especially notable at specific dosage levels.

### 2.8. KEGG Pathway Analysis

**Figure 10:**
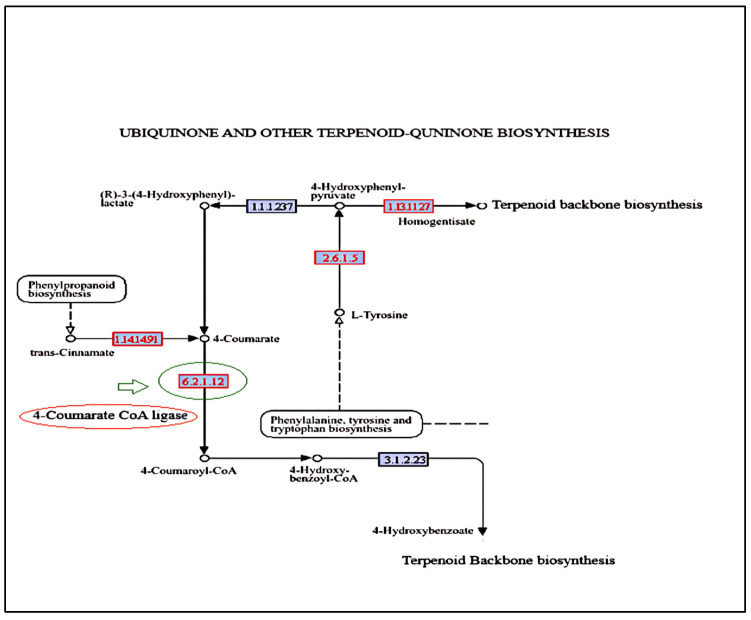
KEGG pathway showing location of 4-coumarate CoA ligase in the terpenoid-ubiquinone pathway. Dotted arrows indicate the multistep biosynthetic pathways. EC 2.6.1.5: Tyrosine aminotransferase; EC 1.13.11.2: 4-hydroxyphenylpyruvate dioxygenase; EC 1.1.1.2.37: hydroxyphenylpyruvate reductase; EC1.14.14.91: cinnamate4-hydroxylase; EC 6.2.1.12: 4-coumarte CoA ligase; EC 3.1.2.23: 4-hydroxybenzoyl-CoA thioesterase.

The KEGG pathway analysis (Figure 10) revealed downregulated and upregulated genes. The ubiquinone and terpenoid-quinone biosynthesis pathway highlights polyprenoid biosynthesis involving the main enzyme 4-coumarate Co A ligase (EC. 6.2.1.12) encoded by the unigene _33114 (Figure 10). The end products of the phenylpropanoid biochemical pathway, such as flavonoids and lignins, have carbon influx directed by this enzyme, where 4-coumaric acid and its hydroxylated derivatives are converted into CoA thiol esters (Ghatak et al. 2023) [23]. Although 4-coumarate CoA ligase does not directly contribute to nitrogen metabolism, its role in the biosynthesis of phenylpropanoid can indirectly influence nitrogen metabolism by affecting resource distribution, stress response, and interactions with other metabolic pathways.

### 2.9. Transcription Factors Identified

About 28,296 SSR’s were identified for the unigene sequences of each sample using the MISA perl script. The predicted unigenes underwent similarity searches against the database specifically meant for plant transcription factors (https://planttfdb.gao-lab.org/) using BLASTP with an e-value threshold of 10^−5^. Hits were obtained against 70267 unigenes. The top 10 most abundant TF families identified are indicated in Table 4 below.

### 2.10. Validation of Plants with Genome-Specific DArT-Based Marker and Other Identified SSR Markers

**Figure 11:**
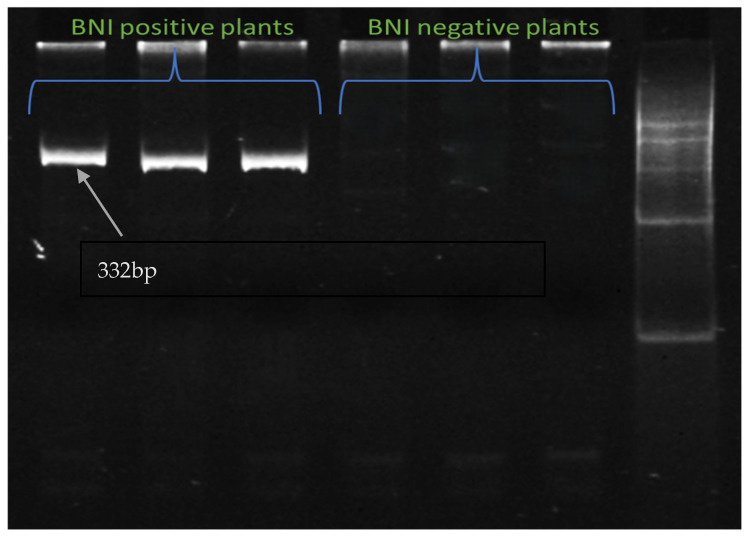
Polyacrylamide gel image showing amplification of introgressed segment with genome-based marker *24_s81287*, where BNI-positive plants have bands, while BNI-negative plants have no bands.

A Genome-specific Diversity-array Technology (DArT)-based marker was developed to detect the introgression of *Leymus racemosus* into the short arm of the 3B chromosome of wheat. This short introgressed segment contains the BNI trait. Some BNI positive plant and BNI negative plants from the field had their DNA extracted and this genome specific marker was run on them to check the introgression. Figure 11 shows the presence of the BNI trait in BNI positive plants while BNI-negative plants show no amplification.

**Figure 12:**
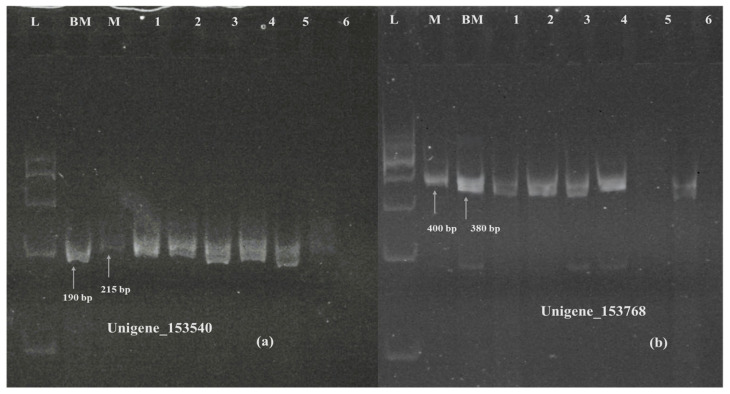
Polyacrylamide gel image showing amplification of SSR marker validation of downregulated Unigene_153540 (**a**) and Unigene_153768 (**b**). M: Munal; BM: BNI Munal; L: 100 bp ladder; Lane 1 to 6: BNI positive lines.

**Figure 13:**
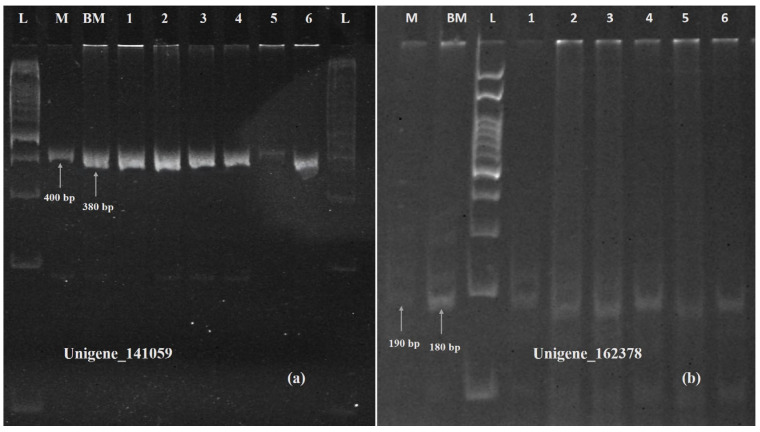
Polyacrylamide gel image showing amplification of SSR marker validation of upregulated Unigene_141059 (**a**) and Unigene_162379 (**b**). M: Munal; BM: BNI Munal; L: 100 bp ladder; Lane 1 to 6: BNI positive lines.

Six SSR primers, five for unigenes showing differential expression and one for a unigene highlighted in KEGG pathway analysis, were designed using the Primer3Plus online program, which was later validated on polyacrylamide gel electrophoresis (Supplementary Materials Table S4). The primers designed for unigenes 153768 (TaVRN-A1), 153540 (NRT1) (Figure 12), 141059 (PPR containing protein), and Unigene_162379 (cytokinin dehydrogenase) (Figure 13) showed polymorphism between Munal and BNI-Munal, except for Unigene_222562 (NrpA gene) and Unigene_33114 (4-coumarate CoA ligase), which did not show any polymorphism between Munal and BNI-Munal. The following markers were also validated on 10 BNI-positive individual plants selected from the F6 generation of cross DBW222 × BNI-Munal gel images, which are shown in Figure 11.

## 3. Discussion

Transcriptomics is a valuable tool for understanding gene expressions related to nitrogen assimilation and metabolism at the RNA level [24]. It can elucidate the molecular basis of BNI by identifying genes responsible for root exudation of nitrogen-inhibiting compounds, and it can also identify regulatory networks and transcription factors controlling nitrogen fixation [21,25]. Modulating gene expression under different nitrogen doses can aid breeders in selecting stress-responsive genes for elite wheat cultivars and in discovering novel genes and pathways involved in nitrogen fixation [26]. Furthermore, transcriptomics can reveal the impact of varying nitrogen doses on other nutrient cycles [27] and provide sequence information for developing molecular markers (SSRs and SNPs) to improve nitrogen uptake, assimilation, and metabolism in wheat.

This study investigated Munal and BNI-Munal wheat genotypes under varying nitrogen levels to identify genes linked to efficient nitrogen metabolism and NUE. Unigene prediction was employed to identify unique gene sequences from RNA-Seq data, aiding gene discovery and annotation. The analysis predicted numerous unigenes, with three showing upregulation and two showing downregulation across all nitrogen regimes. Functional annotation via BLAST search on the NCBI website revealed insights into the potential roles of these genes.

The upregulated unigene Transcript_222562 aligned with the NrpA gene in the *T. aestivum* strain CRB-INFRA-CFD-3358, encoding a nodulin-related protein involved in nitrogen regulation in archaebacteria [28] In wheat, similar genes relate to nitrogen fixation and NUE, with nodulin-like proteins playing roles in nutrient transport and allocation, impacting NUE [29,30]. The upregulated unigenes Transcript_141059 and Transcript_162379 were identified as encoding a *pentatricopeptide repeat-containing protein* (PPR) and *cytokinin dehydrogenase 2*, respectively in *T. aestivum*. The PPR proteins, primarily in mitochondria, maintain respiratory pathways crucial for nitrogen assimilation. Disruption of PPR gene function has been shown to impair nitrogen utilization in plants, particularly under stress conditions. Efficient PPR function is, therefore, considered vital for optimal NUE [31]. Cytokinin dehydrogenase 2 (CKX2) is a key regulator of plant growth and development, affecting root architecture and nitrogen uptake efficiency [28]. The *CKX2* gene in bread wheat is present on chromosomes 3A, 3B, and 3D. The *TaCKX2.2.1* gene on chromosome 3A influences seed size, while the ortholog on 3D affects seed number. Further research is required to determine whether the SSR marker developed in this study specifically identifies the *TaCKX2.2.1* gene on chromosomes 3A, 3B, or 3D.

Among the downregulated unigenes, Transcript_153540 aligned significantly with the *Triticum aestivum* 3BS-specific BAC library. This library’s coding sequence matched the NRT1/PTR (Nitrate Transporter/Peptide Transporter) family, indicating a shift in nitrogen uptake from nitrate to nitrite due to BNI activity [32,33] This explains why the nitrate transporter proteins are significantly downregulated in the current study.

The Nitrate Transporter (NRT) superfamily of proteins regulates the transport from roots to the developing parts of the plant, i.e., the sink. The NRT1 family is involved in nitrogen uptake from the soil into all the developing parts of the plant [34]. NRT2.1, 2.2, 2.4, and 2.5 dominate nitrate uptake in deficient conditions because of high affinity [33]. NRT1.7 facilitates the re-mobilization of nitrates from older leaves to developing younger leaves. NRT1.9 facilitates the transport of nitrates between the xylem and phloem [35]. The NRT1.13 [36], NRT1.11, and NRT1.12 [37] transporters facilitate nitrate accumulation in vacuoles for storage and future use. This prepares the plant to face nitrate stress conditions. Later, these nitrates can be transported to the sink, such as seeds. The SSR marker developed in this study could not identify which NRT gene it is associated with, which can be determined if studied further. However, the significant downregulation of nitrate transporter genes is consistent with previous reports in wheat grown under varying nitrate and ammonium fertilizer regimes [38].

Another downregulated unigene, Transcript_153768, aligned with the *TaVRN* gene family, is associated with vernalization and flowering time regulation. The *TaVRN-A1* and *TaANR1* genes, linked to NUE, were downregulated under nitrogen availability, aligning with BNI-Munal’s nitrate inhibition [39,40]. Favorable alleles of these genes could enhance yield, and molecular markers developed from them may aid in breeding elite cultivars with improved NUE. *TaHOX1*, encoding a *Triticum aestivum* Homeobox 1 protein, is another key gene involved in development in response to environmental factors, such as nutrient availability. The interaction of *TaVRN1*, *TaANR1*, and *TaHOX1* optimizes root growth for nitrogen uptake and these could be important genes to consider during the selection process [41].

One of the unigenes, 33114, encodes 4-courmarte CoA ligase, which is responsible for the ligation of CoA to 4-coumaric acid to change it into the intermediate product called 4-coumaroyl CoA in the quinone terpenoid-quinone pathway. Nucleotide BLAST analysis of the transcript sequence was able to provide a hit against the glutathione metabolism pathway. This study draws parallel to another investigation undertaken by [38], where KEGG pathway analysis indicated that glutathione metabolism and phenylpropanoid biosynthesis were the main enriched pathways. These pathways are responsible for the synthesis of secondary metabolites like plastoquinone and ubiquinone. Terpenoid-quinone-derived molecules, like plastoquinones, are crucial for the functionality of *nitrate reductase* (*NR*) and *nitrite reductase* (*NiR*) enzymes in nitrogen assimilation. Improved activity of these enzymes enhances NUE by promoting the incorporation of nitrogen into amino acids and proteins [42]. In crops like sorghum and rice, secondary metabolites like sorgholeone exhibit BNI activity via root exudation, adapting to soil pH and nutrient conditions [20,43]. Sorgholeone’s exudation into the soil occurs through exocytosis, influenced by the rhizosphere’s chemical environment.

The PPR protein ensures mitochondrial energy production, which powers NRT2-mediated nitrate transport [44,45]. Cytokinin dehydrogenase fine-tunes the root architecture, modulating NRT2 efficiency in nitrate uptake [45,46]. Whereas NrpA mitigates the impacts of stress on nitrogen metabolism, NRT2 transporters adjust uptake in response to phosphorus availability via *NIGT1.2* [47]. VRN-A1 potentially synchronizes nitrogen utilization with growth stages, optimizing NUE during critical phases, such as flowering. These genes form a network where PPR proteins sustain energy metabolism, cytokinin dehydrogenase balances root-shoot allocation, NRT2 transporters directly mediate nitrogen uptake, NrpA buffers environmental stresses, and VRN-A1 optimizes developmental timing. Their combined activities enhance NUE by improving nitrogen acquisition, assimilation, and remobilization under varying conditions.

In conclusion, this study highlights the potential of BNI-Munal as a valuable resource for breeding more nitrogen-efficient wheat genotypes. The identified genes and pathways provide targets for future research to improve nitrogen use efficiency in wheat and promote sustainable agricultural practices.

## 4. Materials and Methods

### 4.1. Plant Material, Nitrogen Dose Administration, and Data Collection of Agro-Morphological Traits

**Figure 14:**
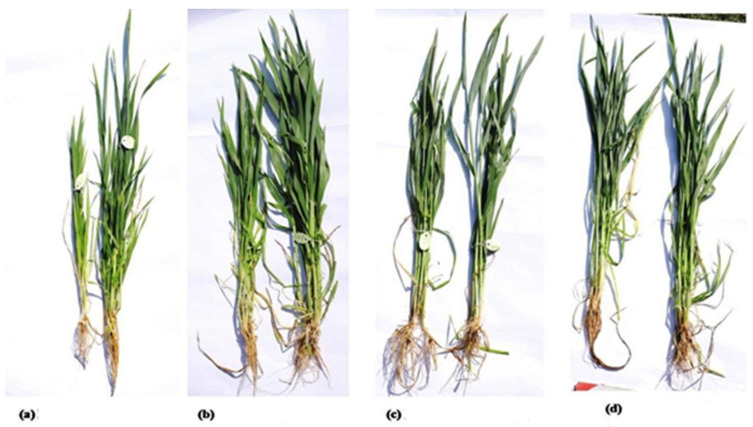
Differences in plant morphology (Munal and BNI Munal parents) at different nitrogen management levels, viz., 0% N (**a**), 50% N (**b**), 75% N (**c**), and 100% N (**d**).

This study utilized two cultivars of wheat (*Triticum aestivum*), Munal, identified as nitrogen inefficient, and BNI-Munal (CS MONO 3B//CS*2/LE RA/3/CS PH/4/6*MUNAL), known for its efficiency under low nitrogen input (Figure 14). A segment of chromosome (Lr#3Ns) from the perennial Mammoth grass (*Leymus racemosus*) was introgressed as a 3BS specific translocation (T3BL.3Nsb S) into *T. aestivum* cv. Munal, now named BNI-Munal [12]. *Leymus racemosus* exhibits higher NUE as compared to normal wheat cultivars due to the ability of its roots to produce biological nitrogen inhibitors, reducing soil nitrifying activity and subsequent nitrate leaching [48].

The field experiment was conducted in the first week of November (4 November 2024) during the wheat crop season 2023-24. The two cultivars (Munal and BNI Munal) were grown under four different nitrogen management levels: 0%, 50% (75:60:40::N:P:K), 75% (112:60:40::N:P:K), and 100% (150:60:40::N:P:K) in a randomized block design pattern with two replications under each level. Morphological data for grain yield and related traits like plant height (cm), flag leaf length (cm), 1000-grain weight (g), tillers per meter, and %protein content were as per [49]. The data were recorded for two consecutive years, viz., 2023 and 2024. Statistical analysis of these traits was performed using RStudio software (package Agricolae) [50].

### 4.2. Sample Collection for Transcriptomic Analysis

Using an in-house RNA isolation method described by [51], flag leaf and second leaf samples were collected in labeled 50 mL falcon tubes at Zadok growth scale 30–45 days crop [52] in the first week of January. These samples were quickly frozen in liquid nitrogen and stored at −80 °C for later RNA extraction.

### 4.3. RNA Isolation and Nitrogen Management Level

For RNA extraction, eight samples were selected from the entire flag leaf and a second leaf of two wheat cultivars, Munal and BNI Munal. These samples were treated with different doses of nitrogen. For Munal, treatments were labeled as T1(0%), T3(75:60:40::N:P:K), T5(112:60:40::N:P:K), and T7(150:60:40::N:P:K)}, and for BNI–Munal, treatments were named as T2(0%), T4(75:60:40::N:P:K), T6(112:60:40::N:P:K, and T8(150:60:40::N:P:K)} (Table 5).

### 4.4. Library Construction and Sequencing

The construction of the cDNA library and Illumina sequencing were conducted using the Illumina Novaseq 6000 (Illumina Inc., San Diego, CA, USA) for cluster generation, in collaboration with UNIGENOME: Leading Genomics Innovation. RNA integrity was confirmed using an Agilent 4150 tape station (Agilent Technologies, Palo Alto, CA, USA).

By using oligo (dT) beads the polyadenylated mRNA was isolated and subsequently broken into fragments using a specialized fragmentation buffer. The first strand of complementary DNA (cDNA) was synthesized using random hexamers and reverse transcriptase enzyme. The second strand synthesis was performed using a specialized buffer provided by Illumina, dNTPs, RNase H, and *Escherichia coli* DNA polymerase I through a nick-translation mechanism. The resulting cDNA library was finalized through sequential processes, including steps like purification, end repair, A-tail addition, adapter ligation, size selection, and finally, PCR amplification. The library was combined with other samples, denatured and loaded on to the flow cell, followed by its sequencing using the Illumina Novaseq 6000 system. The paired-end sequencing library was prepared using a KAPA mRNA HyperPrep Kit for Illumina by Illumina Inc., San Diego, CA, USA (CAT #KK8581). The adapters are designed to allow selective cleavage of the forward strands after re-synthesis of the reverse strand during sequencing. The copied reverse strand was then used to sequence from the opposite end of the fragment. The amplified libraries were analyzed on TapeStation 4150 (Agilent Technologies) using HSD100 ScreenTape^®^.

### 4.5. De Novo RNA-Seq Assembly and High-Quality Read Generation

The de novo assembly of RNA-Seq data was performed using the software Trinity version 2.14.0, followed by downstream analysis [13]. The International Wheat Genome Sequence Consortium referenced genome of cultivar Chinese Spring (denoted as IWGSC CS RefSeq v2.1) was employed for comparative analysis with the de novo unigenes generated. Trim Galore version 0.6.4 was used to filter the raw data, removing adaptor sequences and low-quality bases. This process resulted in high-quality (HQ) clean data, prepared with 500–1000 ng of RNA input for subsequent downstream analyses.

### 4.6. Differential Gene Expression Analysis

The R package ‘edgeR’ was employed to infer differences in gene expression among the samples. To facilitate annotation and sample differential expression analysis, a master assembly was conducted using Trinity (kmer 25) with high-quality adapter trimmed reads from all samples. The CD-HIT program (version 4.8.1) was used for transcripts clustering to predict unigenes, eliminating shorter redundant transcripts that were fully covered by other transcripts with over 90% identity using the CD-HIT-EST program. The resulting non-redundant clustered transcripts were designated as unigenes. To find similarities, a BLASTP search with an e-value cutoff of 10^−5^ was performed, comparing the unigenes against NCBI’s non-redundant (nr) database. Additionally, all unigene sequences were analyzed for similarity to Uniprot, KOG, and Pfam. A Venn diagram was created to illustrate the comparative account of unigene annotation across several databases.

### 4.7. Functional Gene Annotation and Gene Ontology (GO) Term Analysis

Using Blast2GO, the GO terms were mapped for each functionally annotated protein from BLASTP against the NR database. The GO enrichment analysis was performed to identify statistically overrepresented GO terms within the given set of genes. This was achieved through Fisher’s Exact Test, and Benjamini Hochberg’s method was used to control the false discovery rate (FDR). The enriched GO terms were then later examined to identify pertinent biological processes, molecular functions or cellular components.

The KEGG Automatic Annotation Server (KAAS) was used to map the unigenes to the biological pathways and assign orthologs. All unigenes were compared to the KEGG database using BLASTP with a default cutoff bit-score value of 60. The biochemical pathways, including those for carbohydrates, lipids, nucleotides, amino acids, glycans, cofactors, vitamins, terpenoids, and polyketides, were represented by all unigenes mapped to the KEGG database. These pathways were detected in the unigene sequences of the sample using the MISA Perl script. Additionally, all predicted unigenes were compared to the Plant Transcription Factor Database (PlantTFDB) using BLASTP with an e-value cutoff of 10^−5^ (PlantTFDB) (https://planttfdb.gao-lab.org/).

Annotation of DEGs was performed keeping wheat genome IWGSC release 2.1 as reference. Volcano plots were generated to illustrate observed differences in gene expression. The co-upregulated and co-downregulated DEGs were visualized with the help of Venn diagrams. Heat maps generated from log2 fold change data were used to visualize the expression patterns of DEGs, with twenty-five downregulated and twenty-five upregulated transcripts chosen for heatmapping using the ‘pheatmap’ package in RStudio 4.4.2.

### 4.8. Enrichment Analysis Based on Gene Ontology Terms

Enrichment analysis was performed to determine which classes or categories of genes were over-represented and under-represented among those differentially expressed under varying levels of nitrogen stress.

### 4.9. KEGG Pathway Analysis

The KEGG analysis was performed including data preparation, pathway enrichment analysis, mapping, and network analysis [53]. The KEGG Automatic Annotation Server (KAAS) was used to map the unigenes to the biological pathways and assign orthologs. Unigenes were distinguished against the KEGG database using protein BLAST with a default cutoff bit-score of 60, representing genes related to genetic information processing, cellular metabolism, and environmental information processing. KEGG pathway analysis was used to determine the pathways associated with DEGs under varying nitrogen levels using their FASTA sequences.

### 4.10. Validation and Polymerase Chain Reaction Analysis

PCR amplification was conducted using the Mini amp thermal cycler system and DreamTaq Master Mix (applied biosystem by Thermo Fisher Scientific). Validation of five differentially expressed co-regulated genes was performed through native polyacrylamide gel electrophoresis (PAGE). The SSR primers for these genes were designed from their transcript sequence using Primer3Plus version 3.3.0 online program and nucleotide BLAST was further performed to check the specificity of the designed primers.

## Figures and Tables

**Table 1:** Analysis of variance showing different variations contributed by each source for all the recorded traits. PH: Plant height; FLL: Flag leaf length; TPM: Tillers per meter; 1000 GW: 1000-grain weight; N: Grain nitrogen level; GY: Grain yield.

Source of Variation	PH	FLL	TPM	1000 GW	N	GY
Genotype	0.099	0.005 *	0.070	0.003 *	0.841	0.447
Treatment	0.001 *	0.005 *	0.000 *	0.878	0.081	0.000 *
Interaction	0.943	0.562	0.734	0.966	0.997	0.960

‘*’ is at 0.05 level of significance.

**Table 2:** Differential gene expression (DGE) statistics obtained at four nitrogen levels using NovaSeq 6000.

DGE Combination	Total DEGs	Down- Regulated	Up- Regulated	Significant Downregulated	Significant Upregulated	Significant DGE
**C1-N (0%)**	55,668	27,550	28,118	73	298	371
**C2-N (50%)**	36,605	18,277	18,328	31	230	261
**C3-N (75%)**	35,517	17,662	17,855	26	277	303
**C4-N (100%)**	52,732	26,339	26,393	290	446	736

**Table 3:** Differential gene expression statistics showing gene IDs, fold counts, and associated *p* and e-values. Positive logFC value signifies upregulation, while negative logFC value signifies downregulation.

Gene	Sequence Description (Accession ID)	logFC	*p*-Value	E-Value
Unigene_141059	PPR protein (AtPPR), pentatricopeptide repeat-containing protein (XP_044339860.1)	3.1	1.13 × 10^−5^	0
Unigene_162379	cytokinin dehydrogenase (XP_044355953.1)	5	1.46 × 10^−10^	0
Unigene_222562	CRB-INRA-CFD-3358 nodulin-related protein (NrpA) (XP_044353808.1)	6.4	8.42 × 10^−15^	1.6
Unigene_153540	NRT1/PTR FAMILY 2.13-like (XP_044353808.1)	−2.9	3.90 × 10^−5^	1.3
Unigene_153768	VRN-A1 (AAW73220.1)	−3.7	4.22 × 10^−7^	2.00 × 10^−177^
Unigene_33114	*Triticum aestivum* Glutathione hydrolase 1-like (XM_044476980.1)	−0.9	0.15	0

**Table 4:** Top 10 transcription factors families identified.

Transcription Factors ID	TF Name	Basic Functions	Number of Unigenes
bHLH	Basic Helix-Loop-Helix	developmental process, cell differentiation, and stress response	4872
WRKY		stress response and hormonal signalling	4071
FAR1	Far-Red Impaired Response 1	regulation of seed development in far-red light	4003
MYB_related	Myeloblastosis-related	cell cycle regulation, stress response and secondary metabolism	3853
NAC	NAM (*No Apical Meristem* from Petunia), ATAF-1/2 (*Arabidopsis Transcription Activation Factor genes*), and CUC2 (Cup-shaped Cotyledon from *Arabidopsis thaliana*)	plant development and defining organ boundary function	3645
ERF	Ethylene Response Factor	regulating gene expression in response to ethylene levels	3255
B3	B3 Domain Transcription Factor	seed development and embryogenesis	2725
C2H2	Cys2-His2 Zinc Finger	act as transcriptional activators and repressors	2483
bZIP	basic Leucine Zipper	stress response and hormonal signalling, and other developmental process	1946
GRAS	Gibberellic Acid Insensitive, Repressor of GA1 and Scarecrow	plant growth, particularly in gibberellin signalling and root development	1901

**Table 5:** Table demonstrating comparative DGE (differential gene expression) between two genotypes, viz., BNI Munal (treated) vs. Munal (control).

S.No.	Nitrogen Management Levels	DGE Comparisons	BNI Munal (Treated)	Munal (Control)
1	0% N (T1 and T2)	C1	T2	T1
2	50% N (T3 and T4)	C2	T4	T3
3	75% N (T5 and T6)	C3	T6	T5
4	100% N (T7 and T8)	C4	T8	T7

## Data Availability

Data are contained within this article.

## Supplementary Materials

The following supporting information can be downloaded at: https://www.mdpi.com/article/10.3390/ijms26104610/s1.

## Abbreviations

The following abbreviations are used in this manuscript:

BNI	Biological nitrogen inhibition
NUE	Nitrogen use efficiency
KEGG	Kyoto Encyclopedia of Genes and Genomes
GO	Gene ontology
