# Supplementary material for: Transcriptomic Analysis to Understand the Nitrogen Stress Response Mechanism in BNI-Enabled Wheat
Source: Int J Mol Sci. 2025 May 12;26(10):4610. doi: 10.3390/ijms26104610 (PMC12111241; doi:10.3390/ijms26104610)
Supplement: Supplementary file 1 [file ijms-26-04610-s001.zip › ijms-3532613-supplementary-figures.pdf]

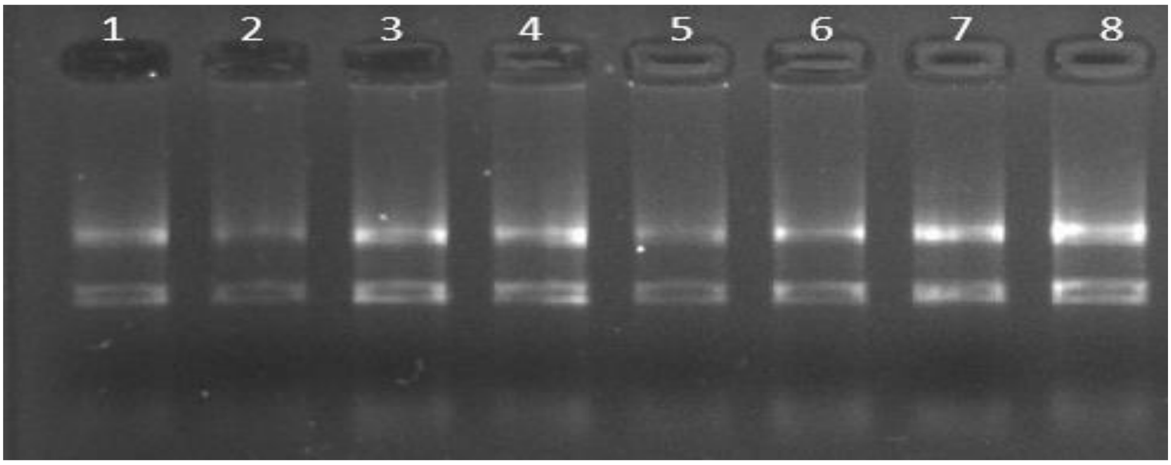

Supplementary Figure S1: RNA qualities check (QC) report on 1% agarose gel

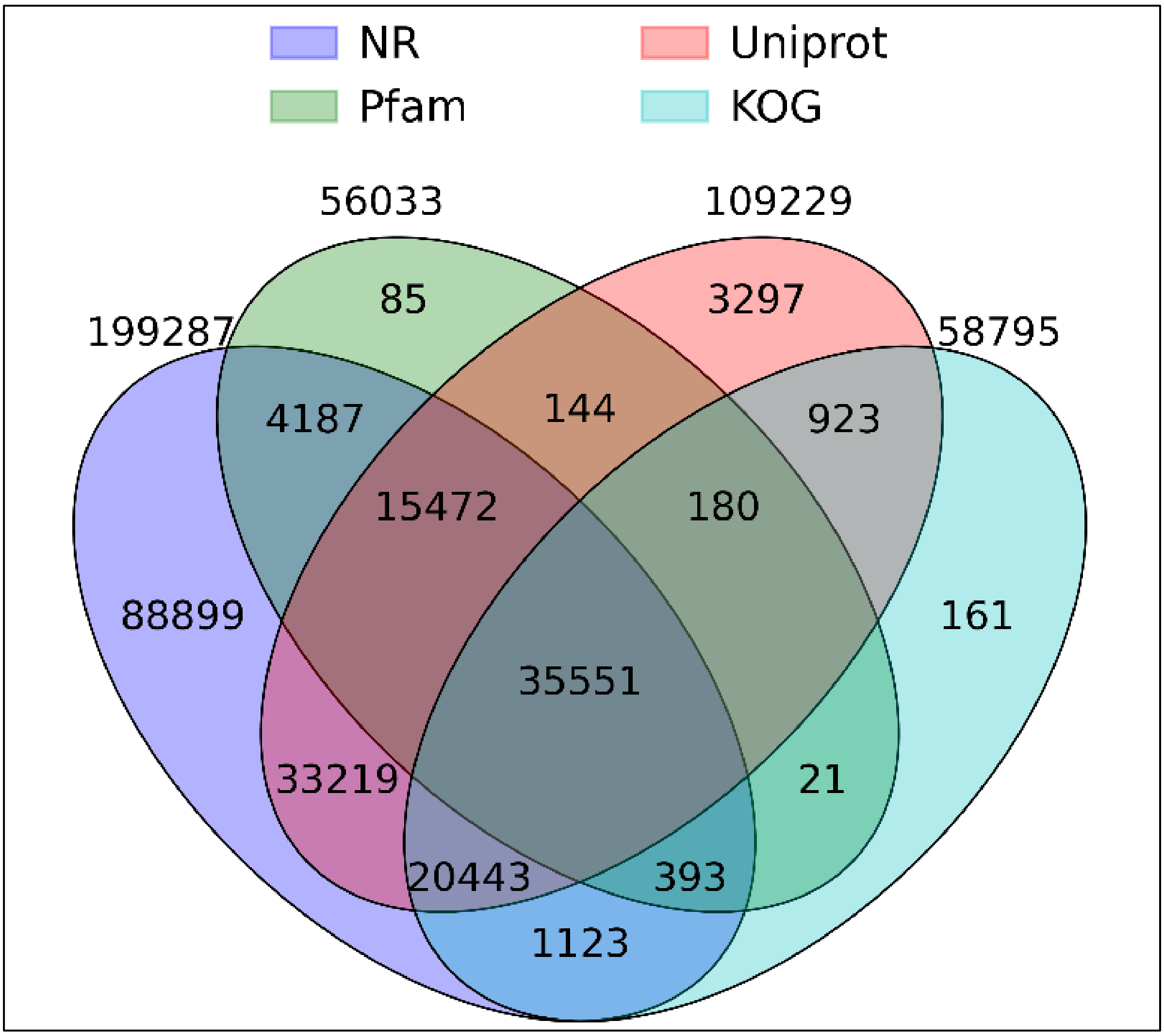

Supplementary Figure S2: Venn diagram for annotated proteins using different databases under different Nitrogen Management level.
