# Supplementary material for: Transcriptomic Analysis to Understand the Nitrogen Stress Response Mechanism in BNI-Enabled Wheat
Source: Int J Mol Sci. 2025 May 12;26(10):4610. doi: 10.3390/ijms26104610 (PMC12111241; doi:10.3390/ijms26104610)
Supplement: Supplementary file 1 [file ijms-26-04610-s001.zip › ijms-3532613-supplementary-tables.pdf]

Supplementary Table S1: Mean agro-morphological data recorded during the years 2023 and 2024 in all the nitrogen management levels.

Genotype	Treatment	Replication	PH	FLL	TPM	TGW	N	GY (q/ha)
Munal	N1	R1	73.27	17.80	74	50.76	1.34	13.47
BNI-Munal	N1	R1	73.50	17.33	47	53.52	1.26	13.75
Munal	N1	R2	71.63	19.87	42	47.68	1.48	12.22
BNI-Munal	N1	R2	81.63	19.80	37	54.96	1.66	25.00
BNI-Munal	N2	R1	106.87	25.23	57	52.92	1.24	32.91
Munal	N2	R1	94.07	18.00	70	51.4	1.28	33.47
BNI-Munal	N2	R2	105.77	24.63	88	58.64	1.46	34.44
Munal	N2	R2	93.70	16.17	70	51.36	1.25	29.02
Munal	N3	R1	89.13	21.23	89	49.08	1.36	37.36
BNI-Munal	N3	R1	105.93	23.30	78	57.56	1.33	43.19
Munal	N3	R2	102.43	16.20	114	51.64	1.33	40.69
BNI-Munal	N3	R2	108.30	22.97	92	59.6	1.60	44.44
BNI-Munal	N4	R1	106.80	22.17	96	55.52	1.31	44.02
Munal	N4	R1	105.07	26.07	134	50.24	1.63	52.36
BNI-Munal	N4	R2	102.97	31.57	98	54.88	1.68	50.27
Munal	N4	R2	104.90	19.63	110	52.16	1.43	55.14

PH: Plant height (cm); FLL: Flag leaf length (cm); TPM: Tillers per meter length; TGW: Thousand Grains Weight (g); N: Nitrogen content in grain; GY: Grain Yield(q/ha)

Supplementary Table S2: Quantification using Qubit 4.0 Fluorometer

Sample ID	Concentration (ng/µl)	Volume (µl)	Yield (µg)	Remarks
**1**	240	50	12	**QC pass**
**2**	488	50	24.4	**QC pass**
**3**	588	50	29.4	**QC pass**
**4**	1021	50	51	**QC pass**
**5**	324	50	16.2	**QC pass**
**6**	830	50	41.5	**QC pass**
**7**	268	50	13.4	**QC pass**
**8**	542	50	27.1	**QC pass**

Supplementary Table S3: Raw data showing reads that were generated after filtering low-quality bases in Illumina Novaseq 6000

Control/Treatment	Total Reads in R1	Total Reads in R2	Total Reads (R1+R2)	Total Bases (R1+R2)	Total Data (GB)
**T1**	31139897	31139897	62279794	9902487246	9.90
**T2**	37560873	37560873	75121746	11944357614	11.94
**T3**	46641016	46641016	93282032	14831843088	14.83
**T4**	47499195	47499195	94998390	15104744010	15.10
**T5**	40745181	40745181	81490362	12956967558	12.96
**T6**	32267703	32267703	64535406	10261129554	10.26
**T7**	32931626	32931626	65863252	10472257068	10.47
**T8**	31704929	31704929	63409858	10082167422	10.08

Supplementary Table S4: Details of SSR primers used for validation

S.No.	Primer name	Sequence	T_a	Amplicon size	Polymorphism present
**1**	153540	F-GGTATACGCCAAGTTCAGAGGCGTTTC<br>R-TCGTCTTGCTGCTCACCTCAATACACT	55	219	Yes
**2**	141059	F-GAGCACTATGTAATGGAGGAAAGGTCAC<br>R-GCTCCTCTTAGAACACTGATAGGAATGC	52	410	Yes
**3**	162379	F-CTGGACCAAAAGAGCGATCAGACTAAG<br>R-CTCTAGCTTTCTGTGGCAACCTCAAGA	52	190	Yes
**4**	222562	F-TCTTCTCAGGGAGGTTCTTCGAAA<br>R-AAATGACCTAACAGTACGGATGGC	50	130	No
**5**	153768	F-GGACAGGGGTGCATAAAAGTTAGC<br>R-TCGACAAGGATAGAGTTCATATGGA	48	340	Yes
**6**	33114	F-TGCTGGTTTTGAACATCCTGGC<br>R-AGACAACCGCACCAACATCAAC	51	150	Yes
